# Efficacy of Primaquine in Preventing Short- and Long-Latency *Plasmodium vivax* Relapses in Nepal

**DOI:** 10.1093/infdis/jiz126

**Published:** 2019-03-18

**Authors:** Komal Raj Rijal, Bipin Adhikari, Prakash Ghimire, Megha Raj Banjara, Garib Das Thakur, Borimas Hanboonkunupakarn, Mallika Imwong, Kesinee Chotivanich, Nicholas P J Day, Nicholas J White, Sasithon Pukrittayakamee

**Affiliations:** 1 Department of Clinical Tropical Medicine, Faculty of Tropical Medicine, Mahidol University, Bangkok, Thailand; 2 Central Department of Microbiology, Tribhuvan University, Kirtipur, Kathmandu, Nepal; 3 Mahidol-Oxford Tropical Medicine Research Unit, Faculty of Tropical Medicine, Mahidol University, Bangkok, Thailand; 4 Centre for Tropical Medicine and Global Health, Nuffield Department of Medicine, University of Oxford, United Kingdom; 5 Ministry of Health and Population, Ramshahpath, Kathmandu, Nepal; 6 Department of Molecular Tropical Medicine and Genetics, Faculty of Tropical Medicine, Mahidol University; 7 The Royal Institute, Grand Palace, Bangkok, Thailand

**Keywords:** *Plasmodium vivax*, relapse, chloroquine, primaquine, Nepal

## Abstract

**Background:**

*Plasmodium vivax* is the main cause of malaria in Nepal. Relapse patterns have not been characterized previously.

**Methods:**

Patients with *P. vivax* malaria were randomized to receive chloroquine (CQ; 25 mg base/kg given over 3 days) alone or together with primaquine (PQ; 0.25 mg base/kg/day for 14 days) and followed intensively for 1 month, then at 1- to 2-month intervals for 1 year. Parasite isolates were genotyped.

**Results:**

One hundred and one (49%) patients received CQ and 105 (51%) received CQ + PQ. In the CQ + PQ arm, there were 3 (4.1%) recurrences in the 73 patients who completed 1 year of follow-up compared with 22 of 78 (28.2%) in the CQ-only arm (risk ratio, 0.146 [95% confidence interval, .046–.467]; *P* < .0001). Microsatellite genotyping showed relatively high *P. vivax* genetic diversity (mean heterozygosity, 0.843 [range 0.570–0.989] with low multiplicity of infection (mean, 1.05) reflecting a low transmission preelimination setting. Of the 12 genetically homologous relapses, 5 (42%) occurred in a cluster after 9 months, indicating long latency.

**Conclusions:**

Although there may be emerging CQ resistance, the combination of CQ and the standard-dose 14-day PQ regimen is highly efficacious in providing radical cure of short- and long-latency *P. vivax* malaria in Nepal.

## Teaser

Chloroquine and a standard-dose 14-day primaquine regimen for *Plasmodium vivax* malaria treatment in Nepal proved to be well tolerated and highly effective in preventing both short- and long-latency relapses.

Malaria is endemic in the southern plain of Nepal (the Terai), which shares a long and porous border with India. Nepal has a target of malaria elimination by 2025 and is currently at the pre-elimination stage [1]. More than 80% of malaria infections in Nepal are caused by the relapsing parasite *Plasmodium vivax* [2, 3]. Elimination efforts are challenged by the survival advantage of *P. vivax* in cooler temperatures, possible long latency with “overwintering” of parasites in the human host from one season to the next [4–6], and the need for deployment of diagnostic tests for glucose-6-phosphate dehydrogenase (G6PD) deficiency to provide radical cure safely with primaquine (PQ) [7, 8]. Currently, the World Health Organization (WHO) recommends treating vivax malaria with either chloroquine (CQ) or artemisinin combination therapy, which are blood schizonticides, plus PQ, a tissue schizonticide that eliminates the persistent liver stages (hypnozoites) and thereby provides radical cure [9]. Chloroquine resistance in *P. vivax* was reported first in Indonesia in 1989 [10]. Since then, CQ therapeutic failures in *P. vivax* malaria have been reported from >23 countries, although only 10 countries have reported true resistance in *P. vivax* [11–13]. In the assessment of radical cure regimens for vivax malaria, patient follow-up is generally either for 4 or 6 weeks as for *P. falciparum* therapeutic assessments, or for 6 months, but these durations are insufficient to capture long-latency relapses, which emerge around 8–9 months after the primary infection [14, 15]. Insufficient follow-up results in incorrect low estimates of relapse rates. Identification of resistance and the assessment of the efficacy of current treatment regimens in terms of relapse, recrudescence, and reinfection are augmented by molecular methods, although genotyping alone does not reliably distinguish between these different events [16–19].

The currently recommended treatment for *P. vivax* in Nepal is CQ (25 mg base/kg body weight over 3 days) and PQ (0.25 mg base/kg body weight daily for 14 days) [20]. There is evidence of reduced susceptibility to CQ in some parts of the Indian subcontinent [13], but its current impact in Nepal is uncertain. In South Asia, at least 3 different relapse patterns have been described [15]. In Nepal, there are no studies with the 1-year follow-up necessary to capture long-latency relapses and thus to assess radical curative efficacy reliably. In a study done in the far western region of Nepal, 17% (23/137) of the patients infected with *P. vivax* malaria had recurrences within 6 months documented after the treatment with CQ alone [21]. Nepal aims to eliminate malaria by 2025 and will need highly effective case management to achieve this. The main objective of this longitudinal prospective study with 1 year of follow-up was to determine the true recurrence pattern following treatment with CQ alone. The trial was designed with the additional objectives of capturing long-latency relapses should they occur, of assessing the radical curative efficacy of the currently recommended radical treatment regimen, and measuring the prevalence of G6PD deficiency in the patients.

## METHODS

This was a prospective longitudinal clinical study with 1 year of follow-up to evaluate the clinical and parasitological responses to the nationally recommended radical treatment regimen for acute vivax malaria. Participants in this study were patients >5 years old with microscopy-confirmed *P. vivax* monoinfections who attended different health centers in 3 malaria-endemic districts (Jhapa in Eastern Nepal, and Kailali and Kanchanpur in Western Nepal). Patients with mixed infections, pregnant women, patients taking regular medications for other diseases, and persons unwilling to provide written informed consent and agree to 1 year’s follow-up were excluded from the study.

### Clinical Procedures

On enrollment (day 0), a complete medical history, body weight, demographic information, and contact details were taken (for follow-up). Blood (3–5 mL) was taken for parasite count, G6PD deficiency assessment, hematocrit level, and parasite genotyping. All patients were screened for G6PD deficiency using a standard qualitative test kit (Care Start G6PD deficiency test kit), following the manufacturer’s instructions [8]. Enrolled patients were randomized into 2 treatment groups using pregenerated computer-based random number allocation. Patients were treated with either 3 days CQ (25 mg base/kg given over 3 days) alone, or 3 days CQ (25 mg/kg given over 3 days) plus PQ (0.25 mg base/kg/day for 14 days) (Remedica Ltd). All patients completed a full course of prescribed CQ and PQ.

Counseling was provided to patients at day 0 about malaria and the potential risks and benefits of the study. Patients were requested to attend follow-up on days 1, 3, 7, 14, 21, and 28 and at 1- to 2-month intervals up to a year. In addition, all patients were advised to attend the health center if they developed fever at any time within the 1-year follow-up. Members of the study team made home visits to patients who missed the scheduled follow-up times for clinical examination and collection of blood samples for parasitological analysis. After completion of 1 year of follow-up, all the *P. vivax* patients who received CQ alone were treated with 14 days of PQ to complete a radical treatment course according to the national malaria treatment protocol of Nepal.

### Microscopy

Parasitemia was monitored according to the WHO method for surveillance and drug efficacy assessment [9]. Parasite counts were performed on Giemsa-stained thick and thin blood films. Thick blood films were examined on days 1, 3, 7, 14, 21, and 28. Parasite densities in thick films were calculated assuming a white blood cell count of 8000 cells/μL. Thick and thin smears were examined separately by 2 trained laboratory technicians at each health facility. If there was a difference between the 2 laboratory technicians in species diagnosis or a >25% difference in parasitemia estimation, the smears were rechecked by another trained technician at the respective district public health office.

### Parasite Genotyping

Blood was taken at each follow-up and stored dried on Whatman 3MM Chr filter paper (catalog number 3030–861) at room temperature in resealable plastic bags containing silica gel desiccant beads. Samples were later brought to the molecular malaria laboratory at the Faculty of Tropical Medicine, Mahidol University, Bangkok, Thailand, for microsatellite genotyping. Genomic DNA was extracted using the QIAamp DNA Mini Kit (Qiagen). Eluted genomic DNA was stored at –20°C until testing [22]. Nine *P. vivax* microsatellite markers (Pv 1.501, 3.27, 3.502 and MS1, MS5, MS6, MS7, MS8, and MS16) were used for genotyping [22, 23]. A seminested polymerase chain reaction (PCR) approach was adopted and all amplification reactions were performed in 10 mM/L Tris-Hydrochloric acid (pH 8.3), 50 mmol/L Potassium Chloride, with 250 nmol/L for each oligonucleotide primer, 2.5 mmol/L Magnesium Chloride, 125 µmol/L of each of the 4 deoxynucleotide triphosphates, and 0.4 U of Taq DNA polymerase (total volume 15 µL). The PCR products were analyzed using an ABI genetic analyzer (Applied Biosystems) and allele size, distribution, frequency of the most common allele, and degree of heterozygosity were calculated to assess genetic diversity. Allele lengths and peak heights were measured and quantified using Gene Scan 500 LIZ software. Multiple alleles per locus were assigned if alleles exceeded 33% of the height of the predominate allele [17, 19]. Three alleles were assessed initially, and if 2 or all 3 were the same, a further 6 were typed. Paired isolates were classified as genetically related (homologous) or unrelated (heterologous) as described below. A homologous recurrence could be either a recrudescence (which, unless a minor population only recrudesced, would be expected to be genetically identical except for intrainfection mutations or copy number changes), or it could be a relapse from the same population of parasites that gave the index infection. In the latter case, the sporozoites giving rise to the primary infection and the reactivated hypnozoite could be either genetically identical or genetically related recombinants. In the development of resistance, early relapse precedes recrudescence, so with low-grade resistance, early relapse is the first manifestation [24]. A recurrence was defined as heterologous if it was statistically improbable that it could be related to the primary infection (ie, the majority of typed alleles were different).

### Data Analysis

The χ^2^ or Fisher exact test and Student *t* test were used to assess proportions and ordinal data, respectively. Kaplan–Meier survival analysis was used to calculate recurrence rates using IBM SPSS Statistics for Windows, version 24.0.

**Ethical Considerations**

Ethical approval for this study was obtained from the Nepal Health Research Council (registration number 146/2015) and the ethics committee of the Faculty of Tropical Medicine, Mahidol University, Bangkok, Thailand.

## RESULTS

**Figure 1.**
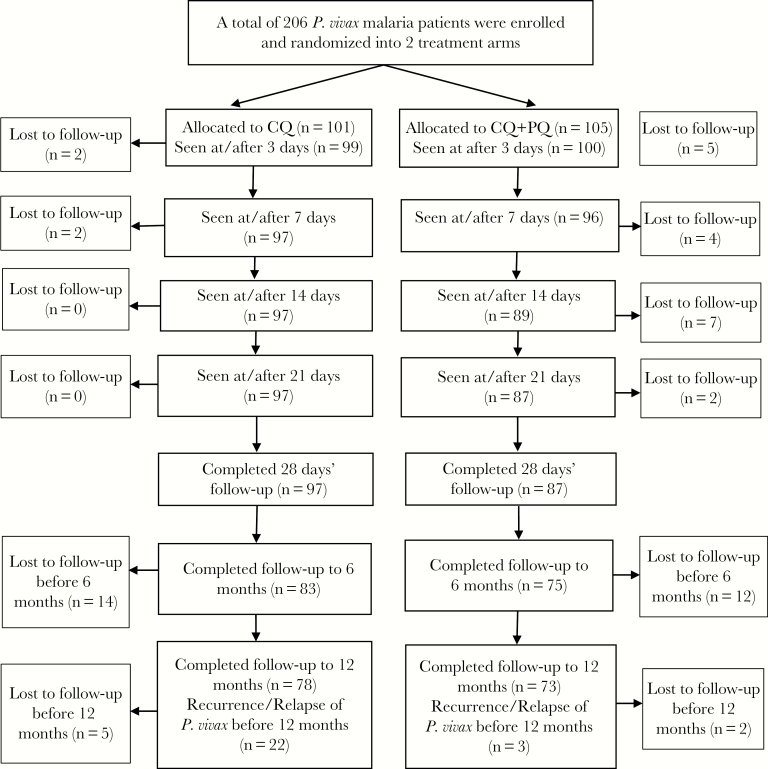
Flowchart of the study. Abbreviations: CQ, chloroquine; PQ, primaquine.

In total, 206 patients were enrolled between December 2015 and December 2017, of whom 101 (49%) were treated with CQ alone and 105 (51%) were treated with CQ + PQ. The majority (147/206 [71.4%]) of *P. vivax* cases were from Kailali district, whereas 26.2% (54/206) were from Kanchanpur district and 2.4% (5/206) were from Jhapa district. Only 2 patients (~1%) were found to be G6PD deficient using the Care Start rapid test kit. Both had been allocated to receive CQ only. The sociodemographic and clinical characteristics of patients were similar in the 2 treatment arms (Table 1). The treatments were generally well tolerated and no severe adverse effects were reported. Of the 206 enrolled cases, 89.3% (184/206) completed the 4-week follow-up schedule of 1, 3, 7, 14, 21, and 28 days (CQ: n = 97; CQ + PQ: n = 87), and 151 (73%) completed follow-up for a full year (CQ: n = 78; CQ + PQ: n = 73) (Figure 1).

**Table 1.** Baseline Characteristics of Study Patients at Enrollment

Characteristic	Treatment Arm			*P* Value
	CQ (n = 101)	CQ + PQ (n = 105)	Total (N = 206)	
District				
Kailali	69 (68.3)	78 (74.3)	147 (71.4)	.51
Kanchanpur	30 (29.7)	24 (22.9)	54 (26.2)	
Jhapa	2 (2.0)	3 (2.9)	5 (2.4)	
Ethnic group				
Janjati	16 (15.8)	17 (16.2)	33 (16.0)	.51
Dalits	39 (38.6)	48 (45.7)	87 (42.2)	
Brahmin and Chhetri	46 (45.5)	40 (38.1)	86 (41.7)	
Male sex	82 (81.2)	88 (83.8)	170 (82.5)	.71
Age, y				
≤17	13 (12.9)	16 (15.2)	29 (14.1)	.58
18–45	80 (79.2)	77 (73.3)	157 (76.2)	
≥46	8 (7.9)	12 (11.4)	20 (9.7)	
Mean (SD); range	27.9 (12.5); 5–75	29.2 (12.5); 6–75	28.5 (13.5); 5–75	
Recent travel outside Nepal	74 (73.3)	86 (81.9)	160 (77.7)	.18
G6PD deficient	2 (2.0)	0 (0.0)	2 (1.0)	
Duration of fever, d, mean (SD)	6.11 (7.80)	5.53 (7.14)	5.82 (7.40)	.22
Weight, kg, median (range)	55 (15–84)	57 (13–75)	55 (13–84)	.27
Temperature, °C, mean (95% CI)	38.8 (36.7–40.6)	38.8 (36.7–40.7)	38.8 (36.7–40.6)	.9
Parasite count, GM (range)	7752 (260–66 400)	8297 (1560–70 800)	8025 (260–70 800)	.38
Hematocrit, %, mean (95% CI)	36.5 (30–46)	36.3 (30–55)	36.4 (30–55)	.28

Data are presented as No. (%) unless otherwise indicated.

Abbreviations: CI, confidence interval; CQ, chloroquine; G6PD, glucose-6-phosphate dehydrogenase; GM, geometric mean; PQ, primaquine; SD, standard deviation.

### Initial Therapeutic Responses

In the CQ arm, 83 (82.2%) patients had persistent parasitemia by day 1 and 17 (17.2%) still had detectable parasitemia by day 3. In the CQ + PQ arm, the corresponding numbers were 88 (83.8%) by day 1 (*P* = .85) and 5 (5.0%) by day 3 (*P* = .006) (Table 2). In the CQ arm, a 33-year-old male patient had persistent parasitemia for 7 days (parasite count on admission was 66 400/µL and at day 7 was 56/µL; ie, early treatment failure), but had cleared by day 14 without further treatment. There was 1 early relapse (at day 21), which was later shown to be genetically heterologous (thereby excluding recrudescence). Apart from these 2 cases, all other patients had adequate clinical and parasitological responses during the 28-day follow-up. In general, the treatments were very well tolerated. Two patients in the CQ + PQ arm were noted to be jaundiced by day 7, but none had severe anemia or black urine.

**Table 2.** Parasitemia Clearance After Treatment and Days Of Relapse

Parasitemia After Treatment	Treatment Arm			*P* Value
	CQ	CQ + PQ	Total	
Day 1 follow-up				
Still parasitemic	83 (82.2)	88 (83.8)	171 (83.0)	.85
Day 3 follow-up				
No parasitemia	82 (82.8.)	95 (95.0)	177 (88.9)	.006
Presence of parasitemia	17 (17.2)	5 (5.0)	22 (11.1)	
Day 7 follow-up				
No parasitemia	96 (99.0)	96 (100)	192 (99.5)	1
Presence of parasitemia	1 (1.0)	0 (0.0)	1 (0.5)	
Days of relapse^a^				
28 days	1 (1.5 [0–3.8])	-(1.2 [0–3.6])	…	
42 days	2 (3.1 [0–6.5])	-(1.2 [0–3.6])	…	
180 days	16 (18.6 [9.9–26.4])	3 (3.9 [0–8.1])	…	
360 days	22 (26.4 [16.2–35.4])	3 (3.9 [0–8.1])	…	

Data are presented as No. (%) unless otherwise indicated.

Abbreviations: CI, confidence interval; CQ, chloroquine; PQ, primaquine.

^a^Data are presented as No. (cumulative % with corresponding Kaplan-Meier estimate [95% CI]).

### Recurrences of *P. vivax* Malaria During 1 Year of Follow-up

**Figure 2.**
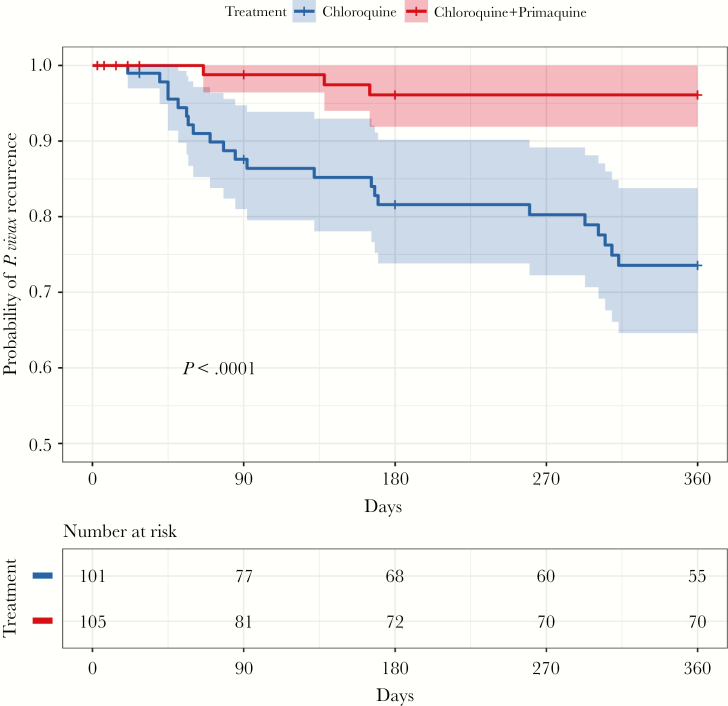
Kaplan–Meier comparison of chloroquine vs chloroquine plus primaquine in the treatment of vivax malaria in Nepal. Shaded areas represent the 95% confidence interval bounds.

In total, 25 (16.6%) patients of the 151 patients who completed follow-up for 1 year had recurrences of vivax malaria (Figure 2). All were symptomatic. Six of the 25 (24%) recurrences occurred after 6 months and occurred in a cluster around 9 months. In the CQ + PQ arm, there were 3 (4.1%) recurrences in the 73 patients followed for 1 year, compared with 22 of 78 (28.2%) in the CQ-only arm (risk ratio, 0.146 [95% confidence interval {CI}, .046–.467]; *P* < .0001. The Kaplan–Meier cumulative mean probabilities of recurrence in the CQ-only group were 1.5% (95% CI, 0–3.8%) at 28 days, 3.1% (95% CI, 0–6.5%) at 42 days, 18.6% (95% CI, 9.9%–26.4%) at 180 days, and 26.4% (95% CI, 16.2%–35.4%) at 1 year. By comparison, in the CQ + PQ group, the cumulative mean probability of recurrence at 1 year was 3.9% (0–8.1%). None of the sociodemographic characteristics were associated with recurrences (supplementary Table 1).

**Figure 3.**
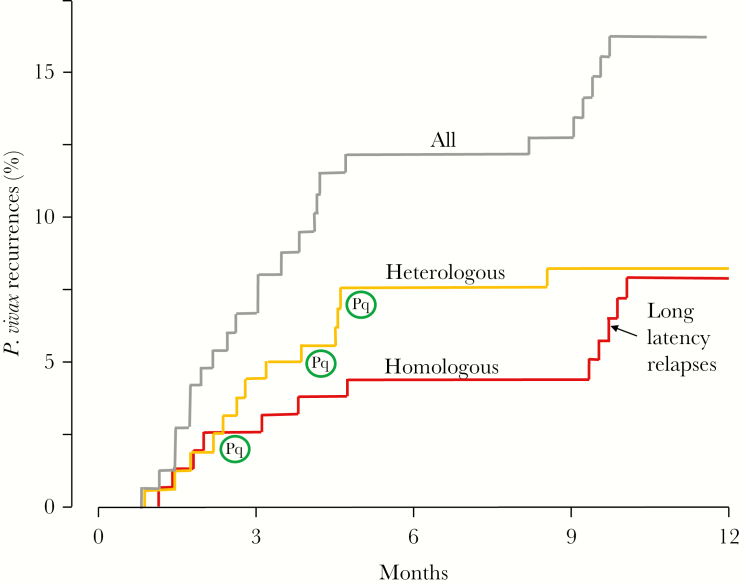
*Plasmodium vivax* recurrences in the 151 patients with a complete 1-year follow-up. Red indicates recurrences with genetically homologous parasites (n = 12) considered as very likely to be relapses. Orange indicates recurrences with genetically unrelated (heterologous) parasites (n = 13), ie, relapses or new infections. Black indicates all the recurrences together. The encircled “Pq” refers to the 3 heterologous recurrences that were in the chloroquine plus primaquine treatment arm. All the others followed chloroquine-only treatment.

There was a high level of diversity among the genetic markers assessed. The highest number of alleles (n = 22) was detected by microsatellite marker MS8 and the lowest number of alleles was found in MS7 (n = 5). The mean heterozygosity of the 9 microsatellite markers in 25 *P. vivax* isolates at day 0 was 0.843 (standard deviation [SD], 0.134). The majority (84%) of infections were apparently of a single genotype. The mean multiplicity of infection was 1.05 (SD, 0.033; range, 1.00–1.08). If all alleles on day 0 (pretreatment) and the day of recurrence were the same, the recurrence was considered definitely to be the same genetically (ie, homologous). Of the 25 paired *P. vivax* isolates, 10 were the same at each locus (ie, identical), 1 pair differed at 1 microsatellite locus, and the other differed at 2 loci. Thus, 12 (48%) recurrences were considered genetically homologous, and therefore assessed as very likely to be relapses. In the other 13 pairs (52%), the majority of alleles were different and so the recurrence isolates were considered heterologous (ie, they could have been genetically heterologous relapses or new infections). Following PQ radical cure, there were only 3 recurrences (by days 66, 138, and 165, respectively) and each was heterologous. As 2 of these occurred >4 months after enrollment, they were considered probable new infections. There was a clear temporal pattern to genetically homologous recurrences (ie, likely relapses), which diverged from heterologous recurrences after 2 months with 7 of 12 relapses occurring within 5 months and the other 5 occurring in a tight cluster after 9 months (Figure 3).

## DISCUSSION

Chloroquine remains the current first-line treatment for vivax malaria in Nepal. In general, CQ is still effective, although one-sixth of patients receiving CQ alone in this study had not cleared their parasitemias by day 3. There was 1 early treatment failure and 1 early relapse (day 21). These findings may well reflect emerging CQ resistance [13]. Adding PQ, which also has asexual stage activity, accelerated parasite clearance significantly and prevented the majority of vivax malaria recurrences. Relapse of vivax malaria is a major cause of morbidity in endemic areas. In this study, with 1 year of follow-up, 28% of the patients who received CQ alone had recurrent vivax malaria. Providing radical cure with PQ reduced recurrences by a factor of 6, from 28.2% to 4.1%. Similar results have been reported in recent years from other sites in this region, from Afghanistan [25], Kolkata, West Bengal, India [26, 27], southwestern India [28, 29], northeastern Myanmar [30], and the Thailand–Myanmar border [23].

Genotyping cannot reliably distinguish relapses from newly acquired infections, as relapses can derive either from hypnozoites derived from sporozoites inoculated by the mosquito that caused the incident infection, or from previously acquired hypnozoites [15, 17]. Overall approximately half the recurrences in this study were genetically homologous (ie, very likely to be definite relapses). Initially the 2 proportions were similar, but after 2 months the temporal pattern clearly diverged with continued heterologous recurrences but no more homologous recurrences until a cluster that emerged after 9 months. This pattern is identical to that observed in studies conducted >10 years ago in Kolkata [31]. Long-latency *P. vivax* is well described from South Asia [14, 15]. Extensive earlier studies characterized the “Madagascar” strain (which, despite its name, may well have originated in India), and also the European, North and Central American, Russian, Central Asian, and Korean long-latency “strains” of *P. vivax* [15]. Long-latency *P. vivax* strains similar to the Madagascar strain may have an early relapse, but more commonly relapse around 9 months after the initial infection [32]. At least 20% of *P. vivax* in Nepal has this phenotype. This emphasizes the necessity of follow-up for 1 year when assessing vivax malaria in areas with long-latency strains. In recent years, this has been shown in Afghanistan [25], Pakistan [33], and Mexico [34], where such strains are prevalent. In this study, at least one-fifth of the relapses would have been missed if follow-up stopped at 6 months.

As in most areas, radical cure with CQ (25 mg base/kg given over 3 days) and PQ (0.25 mg/kg/day for 14 days) was well tolerated and highly effective in Nepal [35]. In this study, the prevalence of G6PD deficiency was very low (2/206 [~1%]) in patients with *P. vivax* malaria. Both the G6PD-deficient patients identified were allocated to the CQ-only group. In nationwide studies conducted among a malaria-uninfected population, the overall prevalence of G6PD deficiency was approximately 6%. However G6PD deficiency in Nepal was more concentrated in particular ethnic groups in the southern plains [7], who were not represented in this study. The prevalence of G6PD deficiency in vivax malaria studies is often lower than in the general population because of the protective effect it confers against *P. vivax* infections [36]. As Nepal is at the preelimination stage, it is important that radical cure be deployed more widely. Availability of G6PD testing would reduce the risks of oxidant hemolysis.

Since 2009, the national malaria control program of Nepal has adopted the 3-day CQ and 14-day PQ regimen as first-line treatment for vivax malaria. As much of the remaining vivax malaria in the country is in the mobile, male migrant labor population, this poses specific challenges for adherence to lengthy treatment regimens such as 14 days of PQ, and consequently to control and elimination. Comprehensive community engagement with all relevant stakeholders, including both private and public healthcare providers, policy makers, health centers, members of the community, and patients attending the clinics, will be needed to ensure success in malaria elimination [37, 38].

This study has a number of limitations. Although this was the first study in Nepal to assess relapse patterns in vivax malaria patients with a follow-up period of a year, supported by parasite genotyping, the number of recurrences was relatively small (n = 25), so the estimates are imprecise. We cannot exclude the possibility of transient asymptomatic recurrences, although this seems unlikely given that all the recurrences identified were symptomatic. Genotyping using 9 polymorphic loci provided satisfactory discrimination, but it does not separate heterologous relapses from new infections. Furthermore, in homologous recurrences, relapse cannot be distinguished with certainty from recrudescences [15, 17]. Using time-to-event information can strengthen the probabilistic discrimination between these states [39]. In this study, recrudescence was considered very unlikely given the low level of CQ resistance. The risks of giving PQ to G6PD-deficient patients in Nepal remain uncertain, although the incidence of G6PD deficiency in vivax malaria patients is low.

In conclusion, this randomized trial confirmed that the currently recommended radical treatment of acute vivax malaria in Nepal with CQ and a standard 14-day course of PQ is highly efficacious in preventing both short- and long-latency relapses despite possible emerging CQ resistance. Long-latency “strains” comprise at least one-fifth of *P. vivax* parasites in Nepal. One year of follow-up is necessary to characterize relapse patterns in this and other areas where these strains are prevalent, otherwise relapse rates will be significantly underestimated.

## Supplementary Data

Supplementary materials are available at *The Journal of Infectious Diseases* online. Consisting of data provided by the authors to benefit the reader, the posted materials are not copyedited and are the sole responsibility of the authors, so questions or comments should be addressed to the corresponding author.

jiz126_suppl_Supplementary_Table_1

## Notes

***Author contributions.* **K. R. R. conducted the field work and drafting of the manuscript. K. R. R., in close supervision of M. I., conducted the molecular work. K. R. R., B. A., and M. R. B. contributed to the statistical analysis and revision of the manuscript. N. J. W., P. G., B. H., M. I., and K. C. reviewed the manuscript. G. D. T. and P. G. provided coordination and implementation of the study. P. G., N. P. J. D., N. J. W., and S. P. contributed to conceptual design and overall method of the study. All authors read and approved the final manuscript.

***Acknowledgments.* **We acknowledge the Epidemiology and Diseases Control Division, Ministry of Health and Population, for its support. We are grateful to Hemraj Joshi, Hari Bhatta, and Bed Ghimire, vector control officers of Kanchanpur, Kailali, and Jhapa districts, respectively, for their help during the entire study period. We thank all the team members and the laboratory technicians of the respective health centers and hospitals for their strong and generous support throughout the study period. We are grateful to Dr James Watson for his contribution in revising the figures in this study, and to all the malaria patients who participated in the study.

***Financial support.* **This research was supported by the Faculty of Tropical Medicine, Mahidol University (Dean’s fund) and by the Mahidol Oxford Research Unit which are supported by the Wellcome Trust. KRR received a PhD scholarship from the Faculty of Tropical Medicine, Mahidol University.

***Potential conflicts of interest.* **All authors: No reported conflicts of interest. All authors have submitted the ICMJE Form for Disclosure of Potential Conflicts of Interest. Conflicts that the editors consider relevant to the content of the manuscript have been disclosed.

## References

[1] Ministry of Health and Population, Government of Nepal. EDCD: Nepal malaria strategic plan 2011–2016 (revised version, December 2011). Kathmandu: Epidemiology and Disease Control Division, Department of Health Services, Ministry of Health and Population, Government of Nepal, 2011.

[2] Rijal KR, Adhikari B, Ghimire P, et al Epidemiology of *Plasmodium vivax* malaria infection in Nepal. Am J Trop Med Hyg 2018; 99:680–7. 30014810 10.4269/ajtmh.18-0373 PMC6169153

[3] Howes RE, Battle KE, Mendis KN, et al Global epidemiology of *Plasmodium vivax*. Am J Trop Med Hyg 2016; 95:15–34. 10.4269/ajtmh.16-0141 PMC5198891 27402513

[4] Battle KE, Gething PW, Elyazar IRF, et al Chapter one–the global public health significance of *Plasmodium vivax*. In: Hay SI, Price R, Baird K, eds. Advances in parasitology. Vol 80 Cambridge, MA: AcademicPress, 2012:1–111. 10.1016/B978-0-12-397900-1.00001-3 23199486

[5] White NJ Why do some primate malarias relapse? Trends Parasitol 2016; 32:918–20. 27743866 10.1016/j.pt.2016.08.014 PMC5134685

[6] Mishra SR, Dhimal M, Guinto RR, Adhikari B, Chu C Threats to malaria elimination in the Himalayas. Lancet Glob Health 2016; 4:e519. 27443775 10.1016/S2214-109X(16)30128-0

[7] Ghimire P, Singh N, Ortega L, et al Glucose-6-phosphate dehydrogenase deficiency in people living in malaria endemic districts of Nepal. Malar J 2017; 16:214. 28535765 10.1186/s12936-017-1864-2 PMC5442674

[8] World Health Organization (WHO). Testing for G6PD deficiency for safe use of primaquine in radical care of P. vivax and P. ovale malaria. Policy brief. Geneva, Switzerland: WHO, 2016.

[9] World Health Organization (WHO). Guidelines for the treatment of malaria. Geneva, Switzerland: WHO, 2015.

[10] Rieckmann KH, Davis DR, Hutton DC *Plasmodium vivax* resistance to chloroquine? Lancet 1989; 2:1183–4. 2572903 10.1016/s0140-6736(89)91792-3

[11] Collignon P Chloroquine resistance in *Plasmodium vivax*. J Infect Dis 1991; 164:222–3. 2056216 10.1093/infdis/164.1.222

[12] Baird JK Chloroquine resistance in *Plasmodium vivax*. Antimicrob Agents Chemother 2004; 48:4075–83. 15504824 10.1128/AAC.48.11.4075-4083.2004 PMC525399

[13] Price RN, von Seidlein L, Valecha N, Nosten F, Baird JK, White NJ Global extent of chloroquine-resistant *Plasmodium vivax*: a systematic review and meta-analysis. Lancet Infect Dis 2014; 14:982–91. 25213732 10.1016/S1473-3099(14)70855-2 PMC4178238

[14] Yorke W Further observations on malaria made during the treatment of general paralysis. Trans R Soc Trop Med Hyg 1925; 19:108–22.

[15] White NJ Determinants of relapse periodicity in *Plasmodium vivax* malaria. Malar J 2011; 10:297. 21989376 10.1186/1475-2875-10-297 PMC3228849

[16] Hwang J, Alemayehu BH, Reithinger R, et al In vivo efficacy of artemether-lumefantrine and chloroquine against *Plasmodium vivax*: a randomized open label trial in central Ethiopia. PLoS One 2013; 8:e63433. 23717423 10.1371/journal.pone.0063433 PMC3661577

[17] Imwong M, Snounou G, Pukrittayakamee S, et al Relapses of *Plasmodium vivax* infection usually result from activation of heterologous hypnozoites. J Infect Dis 2007; 195:927–33. 17330781 10.1086/512241

[18] Chen N, Auliff A, Rieckmann K, Gatton M, Cheng Q Relapses of *Plasmodium vivax* infection result from clonal hypnozoites activated at predetermined intervals. J Infect Dis 2007; 195:934–41. 17330782 10.1086/512242

[19] Imwong M, Sudimack D, Pukrittayakamee S, et al Microsatellite variation, repeat array length, and population history of *Plasmodium vivax*. Mol Biol Evol 2006; 23:1016–8. 16507919 10.1093/molbev/msj116

[20] Epidemiology and Diseases Control Division, Department of Health Services. National malaria treatment protocol—2015. Kathmandu: Epidemiology and Diseases Control Division. Department of Health Services, Government of Nepal, 2015.

[21] Manandhar S, Bhusal CL, Ghimire U, Singh SP, Karmacharya DB, Dixit SM A study on relapse/re-infection rate of *Plasmodium vivax* malaria and identification of the predominant genotypes of *P. vivax* in two endemic districts of Nepal. Malar J 2013; 12:324. 24041296 10.1186/1475-2875-12-324 PMC3848640

[22] Imwong M, Pukrittayakamee S, Gruner AC, et al Practical PCR genotyping protocols for *Plasmodium vivax* using Pvcs and Pvmsp1. Malaria J 2005; 4:20. 10.1186/1475-2875-4-20 PMC1131918 15854233

[23] Chu CS, Phyo AP, Lwin KM, et al Comparison of the cumulative efficacy and safety of chloroquine, artesunate, and chloroquine-primaquine in *Plasmodium vivax* malaria. Clin Infect Dis 2018; 67:1543–9. 29889239 10.1093/cid/ciy319 PMC6206118

[24] White NJ The assessment of antimalarial drug efficacy. Trends Parasitol 2002; 18:458–64. 12377597 10.1016/s1471-4922(02)02373-5

[25] Awab GR, Imwong M, Bancone G, et al Chloroquine-primaquine versus chloroquine alone to treat vivax malaria in Afghanistan: an open randomized superiority trial. Am J Trop Med Hyg 2017; 97:1782–7. 29141719 10.4269/ajtmh.17-0290 PMC5805052

[26] Ganguly S, Saha P, Guha SK, et al In vivo therapeutic efficacy of chloroquine alone or in combination with primaquine against vivax malaria in Kolkata, West Bengal, India, and polymorphism in pvmdr1 and pvcrt-o genes. Antimicrob Agents Chemother 2013; 57:1246–51. 23262997 10.1128/AAC.02050-12 PMC3591881

[27] Ganguly S, Sahaa P, Subhasish G, Basuc N, Majia A Recurrence pattern of *P. vivax* malaria following treatment with chloroquine either alone or in combination with primaquine in urban Kolkata, India. Int J Recent Sci Res 2014; 5:1046–9.

[28] Kumar R, Guddattu V, Saravu K Therapeutic assessment of primaquine for radical cure of *Plasmodium vivax* malaria at primary and tertiary care centres in southwestern India. Korean J Parasitol 2016; 54:733–42. 28095658 10.3347/kjp.2016.54.6.733 PMC5266357

[29] Saravu K, Kumar R, Ashok H, et al Therapeutic assessment of chloroquine-primaquine combined regimen in adult cohort of *Plasmodium vivax* malaria from primary care centres in southwestern India. PLoS One 2016; 11:e0157666. 27315280 10.1371/journal.pone.0157666 PMC4912090

[30] Yuan L, Wang Y, Parker DM, et al Therapeutic responses of *Plasmodium vivax* malaria to chloroquine and primaquine treatment in northeastern Myanmar. Antimicrob Agents Chemother 2015; 59:1230–5. 25512415 10.1128/AAC.04270-14 PMC4335844

[31] Kim JR, Nandy A, Maji AK, et al Genotyping of *Plasmodium vivax* reveals both short and long latency relapse patterns in Kolkata. PLoS One 2012; 7:e39645. 22808048 10.1371/journal.pone.0039645 PMC3396609

[32] James SP, Nicol WD, Shute PG Clinical and parasitological observations on induced malaria. Proc R Soc Med 1936; 29:27–42. 10.1177/003591573602900802 PMC2075988 19990731

[33] Leslie T, Rab MA, Ahmadzai H, et al Compliance with 14-day primaquine therapy for radical cure of vivax malaria—a randomized placebo-controlled trial comparing unsupervised with supervised treatment. Trans R Soc Trop Med Hyg 2004; 98:168–73. 15024927 10.1016/s0035-9203(03)00041-5

[34] Gonzalez-Ceron L, Mu J, Santillán F, et al Molecular and epidemiological characterization of *Plasmodium vivax* recurrent infections in southern Mexico. Parasit Vectors 2013; 6:109. 23597046 10.1186/1756-3305-6-109 PMC3637411

[35] Commons RJ, Simpson JA, Thriemer K, et al The effect of chloroquine dose and primaquine on *Plasmodium vivax* recurrence: a worldwide antimalarial resistance network systematic review and individual patient pooled meta-analysis. Lancet Infect Dis 2018; 18:1025–34. 30033231 10.1016/S1473-3099(18)30348-7 PMC6105624

[36] Louicharoen C, Patin E, Paul R, et al Positively selected G6PD-Mahidol mutation reduces *Plasmodium vivax* density in Southeast Asians. Science 2009; 326:1546–9. 20007901 10.1126/science.1178849

[37] Adhikari B, Pell C, Phommasone K, et al Elements of effective community engagement: lessons from a targeted malaria elimination study in Lao PDR (Laos). Glob Health Action 2017; 10:1366136. 28914184 10.1080/16549716.2017.1366136 PMC5645700

[38] Adhikari B, James N, Newby G, et al Community engagement and population coverage in mass anti-malarial administrations: a systematic literature review. Malar J 2016; 15:523. 27806717 10.1186/s12936-016-1593-y PMC5093999

[39] Taylor AR, Watson J A, Chu C et al Estimating the probable cause of recurrence in *Plasmodium vivax* malaria: relapse, reinfection or recrudescence? BioRxiv 2018 http://biorxiv.org/cgi/content/short/505594v1

