# Supplementary material for: Efficacy of Primaquine in Preventing Short- and Long-Latency Plasmodium vivax Relapses in Nepal
Source: J Infect Dis. 2019 Mar 18;220(3):448–56. doi: 10.1093/infdis/jiz126 (PMC6603971; doi:10.1093/infdis/jiz126)
Supplement: jiz126_suppl_Supplementary_Table_1 [file jiz126_suppl_supplementary_table_1.docx]

| **Supplementary Table 1: Characteristics of study participants in relation to relapse status (n=206)** | | | | |
| --- | --- | --- | --- | --- |
|  | Total | No-relapse | Relapse | p-value |
| Variables | Number (%) | Number (%) | Number (%) |  |
| **Age groups** |  |  |  |  |
| Less than 17 | 29 (14.1) | 26 (14.4) | 3 (12.0) | 0.89 |
| 18-45 years | 157 (76.2) | 137 (75.7) | 20 (80) |  |
| Above 46 years | 20 (9.7) | 18 (9.9) | 2 (8) |  |
| **Gender** |  |  |  |  |
| Male | 170 (82.5) | 152 (84) | 18 (72) | 0.13 |
| Female | 36 (17.5) | 29 (16) | 7 (28) |  |
| **Ethnicity** |  |  |  |  |
| Janjati | 33 (16) | 29 (16.0) | 4 (16.0) | 0.96 |
| Dalits | 87 (42.2) | 77 (42.5) | 10 (40) |  |
| Brahmin and Chhetri | 86 (41.7) | 75 (41.4) | 11 (44) |  |
| **Previous history of malaria** | |  |  |  |
| No | 125 (60.7) | 107 (59.1) | 18 (72.0) | 0.21 |
| Yes | 81 (39.3) | 74 (40.9) | 7 (28.0) |  |
| **Frequency of malaria attacks** | |  |  |  |
| Less or equal to 2 times | 59 (72.8) | 54 (73.0) | 5 (71.4) | 0.93 |
| Above 3 times | 22 (27.2) | 20 (27.0) | 2 (28.6) |  |
| **Family history of malaria** | |  |  |  |
| No | 176 (85.4) | 155(85.6) | 21 (84.0) | 0.82 |
| Yes | 30 (14.6) | 26 (14.4) | 4 (16.0) |  |
| **Travel history outside Nepal** | |  |  |  |
| No | 46 (22.3) | 40 (22.1) | 6 (24.0) | 0.83 |
| Yes | 160 (77.7) | 141 (77.9) | 19 (76.0) |  |
| **History of taking antimalarial** | |  |  |  |
| No | 124 (60.2) | 106 (58.6) | 18 (72.0) | 0.19 |
| Yes | 82 (39.8) | 75 (41.4) | 7 (28.0) |  |
| **Types of malaria** |  |  |  |  |
| Indigenous | 52(25.20 | 45 (24.9) | 7 (28.0) | 0.73 |
| Imported | 154 (74.8) | 136 (75.1) | 18 (72.0) |  |
| **Body temperature °C** |  |  |  |  |
| (Mean± SD) |  | 181(38.82±1.29) | 25 (38.90±1.52) | 0.51* |
| *Independent t- test |  |  |  |  |
